# 

**Published:** 1842-02

**Authors:** 


					﻿THfr
WESTERN JOUMM
OF
MEJJKINE AND SURGEHM
Edited by
fflmEL “DIMKE, M. D.

HaxSReRD^^Y^MbELL,^!. D.
r/>liN WKE LG'UlSVjjI.LIJMMHC AL INSTITUTE,

jM6ma>w. COMWTT, M. D.
. XXV— FEBRE ARY, 1842
A o
LOUISVILLE. KY.
PUBLISHED MONTHLY, BY PRENTICE &. WElbblJNUisK,
PROPRIETORS AdVil <P RENTERS.
0* 0
1842. • ’
This Number contains 4 sheets—Postage under 100 miles 6 cents, over 100 imles 10 cents
				

